# Clinical spectrum of disease and outcomes in children with Omicron SARS-COV-2 infection in Cape Town, South Africa

**DOI:** 10.5588/ijtldopen.23.0053

**Published:** 2024-01-01

**Authors:** C. Bekker, I. Dewandel, A. Redfern, C. McKenzie, J. Lishman, L. M. Verhagen, M. Claassen, S. Wilson, R. Dunbar, C. Bosch, G. van Zyl, W. Preiser, P. Goussard, H. Rabie, M. M. van der Zalm

**Affiliations:** ^1^ Desmond Tutu TB Centre, and; ^2^ Department of Paediatrics and Child Health, Faculty of Medicine and Health Sciences, Stellenbosch University, Cape Town, South Africa; ^3^ Department of Laboratory Medicine, Laboratory of Medical Immunology, Radboud Center for Infectious Diseases; ^4^ Department of Paediatric Infectious Diseases and Immunology, Amalia Children’s Hospital, Radboud University Medical Center, Nijmegen, The Netherlands; ^5^ Division of Medical Virology, Stellenbosch University, Cape Town; ^6^ National Health Laboratory Service (NHLS), Tygerberg, Cape Town, South Africa

**Keywords:** Omicron, tuberculosis, children living with HIV, comorbidities, paediatric

## Abstract

**INTRODUCTION:**

Children with underlying comorbidities and infants are most severely affected by severe acute respiratory syndrome coronavirus-2 (SARS-CoV-2) infection, including in low- and middle-income countries with a high prevalence of HIV and TB. We describe the clinical presentation of SARS-CoV-2 infection in children during the Omicron wave, in Cape Town, South Africa.

**METHODS:**

We analysed routine care data from a prospective cohort of children aged 0–13 years, with a positive SARS-CoV-2 real-time reverse-transcription polymerase chain reaction (rRT-PCR) or SARS-CoV-2 antigen test, admitted to Tygerberg Hospital between 1 November 2021 until 1 March 2022. Risk factors for severity of disease were assessed.

**RESULTS:**

Ninety-five children tested positive for SARS-CoV-2, of whom 87 (91.6%) were symptomatic. Clinical data were available for 86 children. The median age was 11 months (IQR 3.0–60.0), 37 (43.0%) were females, 21 (24.7%) were HIV-exposed and 7 (8.1%) were living with HIV (CLHIV). In total, 44 (51.2%) children had at least one underlying comorbidity. TB co-infection was seen in 11 children, 6 children were newly diagnosed and 5 children were already on TB treatment at the time of admission.

**CONCLUSION:**

There was no evidence of more severe disease in children living with HIV or TB.

## RÉSUMÉ

**INTRODUCTION:**

Les enfants et les nourrissons présentant des comorbidités sous-jacentes sont les plus gravement touchés par l'infection par le coronavirus-2 du syndrome respiratoire aigu sévère (SARS-CoV-2), y compris dans les pays à revenu faible ou intermédiaire où la prévalence du VIH et de la TB est élevée. Nous décrivons la présentation clinique de l'infection par le SARS-CoV-2 chez les enfants pendant la vague Omicron, au Cap, en Afrique du Sud.

**MÉTHODES:**

Nous avons analysé les données de soins de routine d'une cohorte prospective d'enfants âgés de 0 à 13 ans, avec un test positif de réaction en chaîne de la polymérase de transcription inverse en temps réel (rRT-PCR) ou d'antigène du SARS-CoV-2, admis à l'hôpital Tygerberg entre le 1er novembre 2021 et le 1er mars 2022. Les facteurs de risque de gravité de la maladie ont été évalués.

**RÉSULTATS:**

Quatre-vingt-quinze enfants ont été testés positifs au SARS-CoV-2, dont 87 (91,6%) étaient symptomatiques. Des données cliniques étaient disponibles pour 86 enfants. L'âge médian était de 11 mois (IQR 3,0–60,0), 37 (43,0%) étaient des filles, 21 (24,7%) étaient exposés au VIH et 7 (8,1%) vivaient avec le VIH (CLHIV). Au total, 44 (51,2%) enfants présentaient au moins une comorbidité sous-jacente. La co-infection par la TB a été observée chez 11 enfants, 6 enfants ont été nouvellement diagnostiqués et 5 enfants étaient déjà sous traitement antituberculeux au moment de l'admission.

**CONCLUSION:**

Il n'y a pas de preuve d'une maladie plus grave chez les enfants vivant avec le VIH ou la TB.

Omicron (B1.1.529), the fifth variant of concern (VoC) of the severe acute respiratory syndrome coronavirus-2 (SARS-CoV-2), was first reported to the WHO by South African researchers on 24 November 2021.^1,2^ Omicron caused a sharp rise in infections, leading to the fourth peak during the COVID-19 pandemic in South Africa with, for the first time, a decoupling of the number of infections from the numbers of hospitalisations and deaths, especially in adults, likely due to high seroprevalence in the population, either due to natural infection or COVID-19 vaccination resulting in some immunity against the virus.3

Regardless of VoC, the SARS-CoV-2 disease spectrum in children has been described as mild, with mostly out-of-hospital management.^3,4^ However, a differential impact of SARS-CoV-2 was noted in sub-Saharan Africa compared to high-income countries (HICs), with hospitalised children presenting with higher morbidity and mortality.^5–7^ Underlying comorbidities and young age are considered risk factors for more severe disease. Thus, far limited data are available on the role of TB and HIV in severity of COVID-19 disease in children and how this impacted the clinical presentation during the Omicron wave.

The aim of the present study was to describe the clinical presentation and risk factors associated with disease severity in children admitted in a tertiary hospital with SARS-CoV-2 infection during the Omicron wave in Cape Town, South Africa.

## METHODS

This study describes routine care data from children aged 0–13 years with a laboratory-confirmed diagnosis of SARS-CoV-2 presenting to Tygerberg Hospital (TBH) in the period from 1 November 2021 until 1 March 2022. According to the National Institute of Communicable Diseases (NICD) Surveillance report, the Omicron wave in South Africa lasted from 28 November 2021 to 30 January 2022.^8^ We defined laboratory confirmation as a positive real-time reverse-transcription polymerase chain reaction (rRT-PCR) or antigen test for SARS-CoV-2, performed on respiratory samples.

### Setting

The study was conducted in TBH, Cape Town, South Africa, which is an academic teaching hospital that provides secondary-level care to the surrounding geographical areas, as well as tertiary care to the children of the Western Cape Province.^9^ In this resource-limited setting, people experience a high burden of infectious diseases, including TB-HIV and live in poor socio-economic circumstances.^9^ None of the children in this study received a vaccine.

### SARS-CoV-2 molecular testing

Respiratory samples were eluted in phosphate-buffered saline. Total nucleic acid content was isolated using the Nuclisens EasyMag system (bioMerieux, Marcy l’Etoile, France), the NIMBUS automated extraction system (Seegene, Seoul, Republic of Korea), and the MICROLAB^®^ STARlet instrument (Hamilton, Reno, NV, USA) and NucleoMag Pathogen (Macherey–Nagel, Duren, Germany) extraction kit. Positive cases were identified using molecular assays in routine use, including Alinity m SARS-CoV-2 AMP Kit (Abbott Laboratories, Abbott Park, IL, USA), Xpert^®^ Xpress SARS-CoV-2 assay (Cepheid, Sunnyvale, CA, USA), TaqPath COVID-19 CE-IDV RT-PCR assay (Thermo Fisher Scientific, Waltham, MA, USA), Allplex^TM^ 2019-nCoV Assay (Seegene), QA_CFX (Qiagen, Venlo, The Netherlands), and Artus SARS-CoV-2 Prep&Amp UM kit (Qiagen) at the Division of Medical Virology, Stellenbosch University, and the National Health Laboratory Service (NHLS) Tygerberg. Five samples obtained from nasopharyngeal swabs were tested using Abbott Panbio COVID-19 Ag Rapid Test Device (Abbott Diagnostic, Jena, Germany).

### Viral co-infections

Total nucleic acid content was isolated using the Nuclisens EasyMag system, and tested using the Allplex^TM^ Respiratory Panel 1, 2 and 3 Assays (Seegene), which is a multiplex PCR system that detects 16 different viruses in three PCR reactions. These viruses include adenovirus, influenza (A/B) virus, parainfluenza (1-4), human rhinovirus (A/B/C), respiratory syncytial virus (A and B), human bocavirus (1/2/3/4), human metapneumovirus, human coronavirus (229E, NL63, OC43) and human enterovirus.

### Data collection and definitions

Routine care clinicians prospectively completed a case report form for each child with SARS-COV-2 on admission. The hospital laboratory system was used to ensure no children with a positive SARS-COV-2 PCR or antigen test were missed, and we retrospectively completed data of these children from hospital records. Radiological data were collected through the digital site, Picture Archiving and Communication System (PACS). Anterior-posterior (AP) and/or lateral view chest X-ray (CXR) was routinely done in children requiring oxygen or at the discretion of the treating clinician. Single CXR reading was done retrospectively according to the WHO guidelines for pneumonia^10^ by a paediatric pulmonologist (PG).

SARS-CoV-2 testing strategies changed during the pandemic. During the study period, children were only tested if they required admission. Incidental infections were defined as surveillance cases, usually done routinely before planned medical procedures or surgery.

### Severity of disease

We adapted the WHO severity grading for SARS-COV-2 infection in our setting as follows: 1) critical: requiring mechanical ventilation, shock or multi-organ dysfunction/multisystem inflammatory syndrome in children (MIS-C); 2) severe: all children requiring continuous positive airway pressure (CPAP)/high-flow nasal canula (HFNC); 3) moderate: children needing low-flow nasal prong oxygen (NPO_2_) or without oxygen need but with any CXR changes; 4) mild: if symptomatic but without a need for oxygen.

### Data analysis

De-identified data were entered into a RedCap database (Vanderbilt University, Nashville, TN, USA) and analysed using SPSS v27 (IBM SPSS, Armonk, NY, USA).^11,12^ The clinical characteristics of the children with laboratory-confirmed COVID-19 were summarised with descriptive statistics. Pearson χ^2^ test was used to compare the differences between the different age groups. The Yates continuity correction was used if the expected cell size was <5. Non-parametric tests were used to compare differences between the age groups and continuous/dichotomous variables (Kruskal–Wallis test/Mann-Whitney *U*-test). For risk factor analysis, severity of disease was divided into severe disease (critical and severe) and non-severe disease (mild, moderate). Logistic regression was used to analyse risk factors for disease severity using univariate analysis. Variables with *P* < 0.20 were included in the multivariate model.

The Health Research Ethics Committee of Stellenbosch University, South Africa, approved this study (HREC N20/04/013_COVID). The data were entered without patient identifiers using only routinely collected data, a waiver of consent for this process was obtained.

## RESULTS

**Figure 1.**
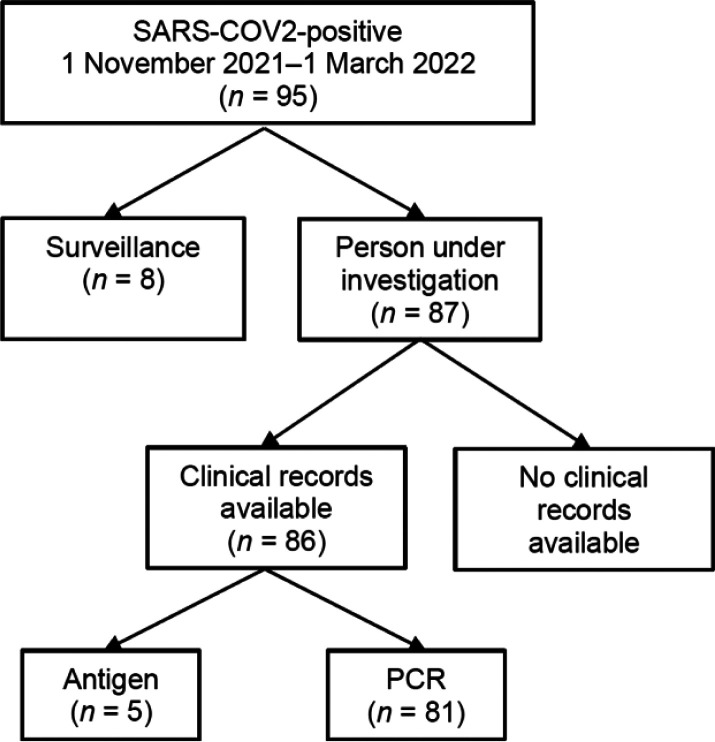
Flowchart of children with SARS-CoV-2 infection during the Omicron wave. In total, 95 SARS-CoV-2 positive RT-PCR and antigen tests were done; 8 were done for surveillance purposes and 87 were persons under investigation. In total, 81 samples were PCR-positive. RT-PCR = reverse transcription polymerase chain reaction.

During the Omicron wave, a total of 95 children tested positive for SARS-CoV-2. In total, there were 87 (91.6%) children with symptoms compatible with COVID-19 and 8 (8.4%) incidental (asymptomatic) detections (Figure 1). Clinical data were lacking in 1 child who was excluded from the analysis, and clinical description was done for the 86 symptomatic cases only. The median age was 11.0 months (interquartile range [IQR] 3.0–60.0); 37/86 (43.0%) were females and 45/86 (52.3%) had one or more underlying comorbidities (Table 1). Prematurity was the most common comorbidity in children under the age of 1 year. Older children more often had a recent diagnosis or history of TB or malignancy. In total, 21/86 (24.7%) children were HIV-exposed, with 7/86 (8.1%) living with HIV (CLHIV).

**Table 1.** Demographics and clinical presentation of SARS-COV-2 positive children by age group.

		All children	0-3 months	3-12 months	1-5 years	≥5 years	
		(n=86)	(n=22)	(n=22)	(n=21)	(n=21)	
	*n*	*n* (%)	*n* (%)	*n* (%)	*n* (%)	*n* (%)	*P* value
Age, months, median [IQR]	86	11 [3.0-60.0]	—	—	—	—	NA
Male sex	86	49.0 (57.0)	11 (50.0)	15 (68.2)	14 (66.7)	19 (90.5)	0.27
Evidence of BCG		58 (67.4)	17 (94.4)	19 (100.0)	12 (85.7)	10 (100.0)	0.24
Comorbidities (any)^*^	86	44 (51.2)	8 (36.4)	13 (59.1)	13 (61.9)	10 (47.6)	0.34
HIV-exposed	85	21 (24.7)	3 (13.6)	8 (36.4)	9 (45.0)	1 (4.8)	0.01
HIV-infected	86	7 (8.1)	0 (0.0)	3 (13.6)	3 (14.3)	1 (4.8)	0.25
TB	86						0.04
Current†		11 (12.8)	0 (0.0)	4 (18.2)	5 (23.8)	2 (9.5)	0.06
Previous		3 (3.5)	0 (0.0)	0 (0.0)	2 (0.5)	1 (4.8)	0.14
Prematurity	49	19 (38.8)	8 (36.4)	7 (31.8)	2 (9.5)	2 (9.5)	0.00
Chronic lung disease	86	1 (1.2)	0 (0.0)	1 (4.5)	0 (0.0)	0 (0.0)	0.05
Cardiac disorder	86	3 (3.5)	1 (4.5)	2 (9.01)	0 (0.0)	0 (0.0)	0.61
Malnutrition	86	6 (7.0)	0 (0.0)	2 (9.1)	3 (14.3)	1 (4.8)	0.26
Malignancy	86	4 (4.7)	0 (0.0)	0 (0.0)	2 (9.5)	2 (9.5)	0.21
Other	86	5 (5.8)	0 (0.0)	0 (0.0)	1 (4.8)	4 (19.0)	0.01
WHO severity grading	86						0.03
Mild		39 (45.3)	8 (27.3)	6 (27.3)	10 (47.8)	17 (81.0)	
Moderate		21 (24.4)	5 (22.7)	8 (36.4)	6 (28.8)	2 (9.5)	
Severe		12 (14.0)	7 (31.8)	4 (18.2)	0 (0.0)	1 (4.8)	
Critical		14 (16.3)	4 (18.2)	4 (18.2)	5 (23.8)	1 (4.8)	
Treatment							
Admission ward	86						0.40
PICU admission		13 (15.1)	6 (27.3)	3 (13.6)	2 (9.5)	2 (9.5)	
Another ward		73 (84.9)	16 (72.7)	19 (86.4)	19 (90.5)	19 (90.5)	
Any respiratory support	86	38 (44.2)	14 (63.6)	16 (72.7)	5 (23.8)	3 (14.3)	<0.01
Highest respiratory support							0.002
Ventilation		9 (10.5)	3 (13.6)	3 (13.6)	2 (9.5)	1 (4.8)	
CPAP		6 (7.0)	4 (18.2)	2 (9.1)	0 (0.0)	0 (0.0)	
HFNC		6 (7.0)	1 (4.5)	4 (18.2)	0 (0.0)	1 (4.8)	
NPO_2_		17 (19.8)	6 (27.3)	7 (31.8)	3 (14.3)	1 (4.8)	
Duration O_2_ required. days, median [IQR]	38	5.0 [1.8-9.0]	5.5 [1.8-10.5]	5.5 [3.0-9.0]	7.0 [1.5-13.0]	5.0 [1-5.0]	0.77
Admission duration, days, median [IQR]	86	6.0 [1.0 11.0]	4.5 [1.0-14.0]	5.5 [3.0-10.3]	8.5 [1.5-14.0]	6.0 [1.0-10.0]	0.42
Study outcome	86						0.17
Discharged home		76 (88.4)	19 (86.4)	22 (100.0)	17 (81.0)	18 (85.7)	
Transferred		7 (8.1)	1 (4.5)	0 (0.0)	4 (19.0)	2 (9.5)	
Died: non-COVID-related		2 (2.3)	1 (4.5)	0 (0.0)	0 (0.0)	1 (4.8)	
Died: COVID-related		1 (1.2)	1 (4.5)	0 (0.0)	0 (0.0)	0 (0.0)	

* Add up to more than 100% as 13 children had more than 1 underlying comorbidity.

† Includes all children with new TB diagnosis on admission (n = 6), as well as children who recently started TB treatment but are still in the intensive phase.

IQR = interquartile range; NA = not available; BCG = acilli Calmette-Guerin; PICU= paediatric intensive care unit; CPAP = continuous positive airway pressure. HFNC = high-flow nasal cannula; NPO_2_ = nasal prong O_2_; O_2_ = oxygen.

The clinical findings varied among the different age groups. Most children presented with fever (31/82, 37.8%), dyspnoea (29/85, 34.1%) or dry cough (23/80, 28.8%). Respiratory symptoms were more frequently seen in younger children. The children aged >5 years presented more often with vomiting (3/20, 15.0%), abnormal neurology (9/20, 45.0%) and seizures (3/20, 15.0%). One 16-month-old child presented with typical febrile seizures. The other eight children with seizures were either very young or older children with a fever and seizure (atypical febrile seizure) or in the typical age group, but without associated fever. None of these children had a previous history of febrile seizures. Two children, both 3 months old, presented with a croup-like illness. One child was ventilated with oxygen requirements for 14 days and the other required CPAP for 3 days.

Laboratory findings of the different age groups were similar. The median C-reactive protein (CRP) of all children was 10 (IQR 3.0–41.8), with the lowest values noted in in the group of children between 1 and 5 years old, and the highest values noted in the oldest age group (≥5 years).

Baseline CXRs were done in 49/86 (57.0%) children, with abnormal findings in 28/49 (57.1%). CXRs were consistent with pneumonia in 22/49 (44.9%) cases and pneumonia with effusion in 2/49 (4.1%) cases. Most children with radiological evidence of pneumonia were less than 1 year old, with 8 (50.0%) who were less than 3 months old.

**Figure 2.**
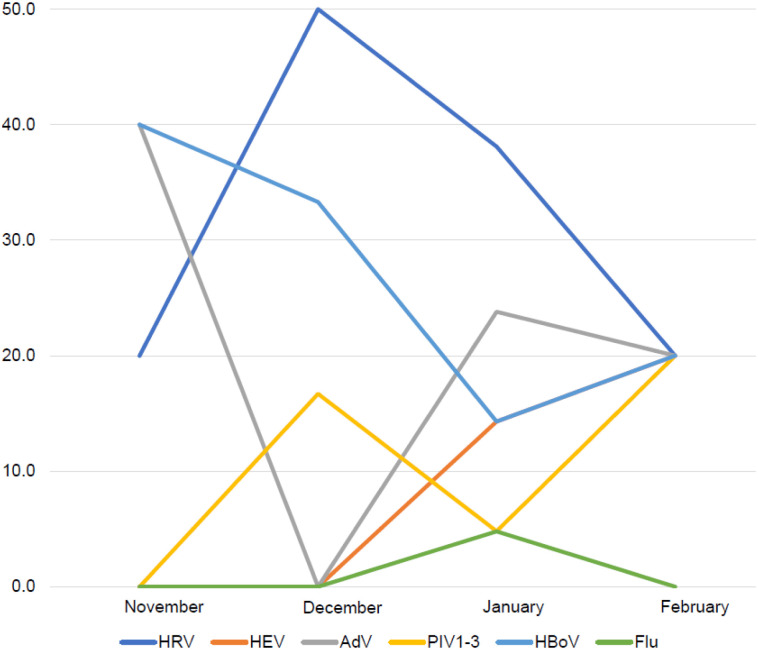
Point prevalence of viral co-infections detected in children with SARS-CoV-2 infection during the Omicron Wave. HRV = human rhinovirus; HEV = human enterovirus; PIV1-3 = para-influenza virus; HboV = human bocavirus; flu = influenza virus A and B.

Nasopharyngeal aspirates (NPAs) were tested for other respiratory viruses in 69/86 (80.2%) children. Of these, 20/69 (29.0%) were positive for one or more additional viruses. Human rhinovirus (11/20, 55.0%), adenovirus (6/20, 30.0%) and human bocavirus (5/20, 25.0%) were

the most frequently detected viral co-infections (Figure 2).

In total, 13 (15.1%) children were admitted to PICU, nine of whom were younger than 1 year of age (*P* = 0.40). Two children were diagnosed with MIS-C, but both were managed outside of the PICU setting. Respiratory support was required in 38 (44.2%) children, with the highest proportion (78.9%) in children younger than 1 year (*P* < 0.01). The overall median oxygen duration need was 5 days (IQR 1.8–9.0), without no observed differences between groups. Eight children, of whom six had at least one comorbidity, required oxygen for ≥10 days.

Using the adapted WHO severity grading system for SARS-COV-2 infection, overall, 14/86 (16.3%) children were assessed as having critical, 12/86 (14.0%) severe and 60/86 (69.8%) non-severe disease (Table 1). Most children (19/26, 73.1%) with critical or severe disease were younger than 1 year. Univariate analysis showed that age, prematurity and features of pneumonia on CXR were associated with more severe disease. In multivariate analysis, this association disappeared (Table 2). HIV exposure, infection or TB disease was not associated with more severe disease.

**Table 2.** Univariate and multivariate logistic regression analysis for severity of COVID-19 disease*

	Univariate analysis			Multivariate analsis		
	OR	95% CI	*P* value	OR	95% CI	*P* value
Age, months	0.99	0.97-1.00	0.03	0.88	0.73-1.07	0.19
Any comorbidities	1.17	0.46-2.93	0.74			
CLHIV	0.36	0.04-3.15	0.36			
HIV-exposed	1.63	0.64-4.21	0.31			
Current TB	0.85	0.21-3.49	0.82			
Prematurity	3.78	0.12-12.8	0.03	6.25	0.82-47.80	0.08
CXR pneumonia	2.94	0.92-9.41	0.07			
Viral co-infection	0.88	0.29-2.71	0.82	3.93	0.55-28.05	0.17

* Only variables that were significant in univariate analysis were included in the multivariate regression.

OR = odds ratio; CI = confidence interval; CLHIV = children living with HIV.

Table 3 shows the clinical presentation and underlying comorbidities seen in children with TB. TB co-infection was seen in 11 children: 6 children were diagnosed with TB during admission and 5 children were recently started on TB treatment. In total, 5 (45.5%) were CLHIV and 4 (36.4%) were confirmed TB, while 7 (63.6%) were clinically diagnosed. Typical features for TB were seen on the CXR of 3/5 (60.0%) children, with hilar lymphadenopathy being the most common. Of the 5 children who were recently diagnosed with TB, 2 had possible TB-immune reconstitution syndrome (IRIS) with clinical and or radiological worsening of disease.

**Table 3.** Clinical description of children with TB and SARS-CoV-2

Case	Age (months)	Reason for admission	Confirmed TB*	New diagnosis	Type	Comorbidities	WHO severity of disease	Highest O_2_ required
#1	4	Severe hypoxic pneumonia	Yes; CXR (expert read): typical TB	Yes	Disseminated TB: pulmonary and abdominal TB	Ex-premature; chronic lung disease	Critical	Ventilation
#2	7	Persistent gastro-enteritis	No; CXR (expert read): typical TB	Yes	Disseminated TB: pulmonary and abdominal-TB	CLHIV; malnutritiona	Moderater	None
#3	8	Cough and growth faltering	No	Yes	PTB	CLHIVA	Severe	HFNC
#4	8	Severe hypoxic pneumonia	Yes	Yes	Disseminated TB: pulmonary and abdominal TB	CLHIV; malnutrition	Mild	None
#5	13	Pneumonia and gleno humeral/sternoclavicular joint TB	Yes	No	Disseminated TB: pulmonary and joint TB	None	Mild	None
#6	14	Severe hypoxic pneumoniao	No	No	PTB	CLHIV	Moderate	NPO_2_
#7	29	SAM	Yes: CXR (expert read): typical TB	Yes	Disseminated TB: pulmonary and spinal TB	SAM	Moderate	NPO_2_
#8	36	Status epilepticus: TBM-IRIS	No	No	Disseminated TB: pulmonary and MDR TBM	None	Mild	None
#9	51	Multisystem inflammatory syndrome in children	No	Yes	PTB	Ex-premature; malnutrition	Critical	None
#10	76	Pneumonia and seizures	No	No	PTB	Cerebral palsy	Moderate	NPO_2_
#11	107	Seizures: TBM-IRIS	No	No	Disseminated: pulmonary and TBM	CLHIV	Mild	None

* Bacteriologically confirmed TB.

O_2_ = oxygen; CXR = chest X-ray; CLHIV = child living with HIV; PTB = pulmonary TB; HFNC = high-flow nasal canula; SAM = severe acute malnutrition; NPO_2_ = nasal prong O_2_; TBM = tuberculous meningitis; IRIS = immune reconstitution syndrome.

The median hospital admission duration was 6 days (IQR 1.0–11.0), without no observed differences between the different age groups. Three (3.5%) children died: one death (1.2%) was considered related to COVID-19. This child was less than 3 months old, and, as previously described, lung tissue was SARS-COV-2-positive at autopsy.^13^ The other two children had comorbidities, one 3-month-old with a cardiac defect and the other almost 9 years old with a pelvic rhabdomyosarcoma complicated by anasarca.

## DISCUSSION

This study describes the clinical presentation and outcomes of children in Cape Town during the Omicron wave. In general, outcomes were good, but more severe disease continued to be seen in infants and young children with underlying comorbidities.

The first Omicron study from Tshwane, South Africa, found that 20.0% of children in their setting required oxygen therapy; however, this was not analysed according to age groups.^14^ Our study found that a third of children aged 0–3 months required some form of respiratory support, which was lower than we previously found during the first SARS-CoV-2 wave in our hospital.^7^ This confirms a trend of less severe SARS-CoV-2 disease seen in our population, with the youngest children remaining at highest risk for severe disease.

Several instances of atypical croup during the Omicron wave have been documented in the literature.^15–18^ These include very young or older children, children with persisting symptoms and those requiring repeated doses of dexamethasone and other steroids.^15,18,19^ In our study, one 3-month-old child required intubation and ventilation for 4 days, and subsequently needed oxygen for 8 more days. The child received a prolonged course of steroids.

Furthermore, a third of children in our study presented with atypical febrile seizures.^20^ A possible explanation for this finding could be that cells within the central nervous system express ACE2, which is a binding site for SARS-CoV-2.^21,22^ A quarter of the children in this cohort were HIV-exposed, with 8% of CLHIV. Previous studies have shown that HIV-exposed, non-infected infants generally have a higher risk of hospitalisation due to a lower respiratory tract infection when compared to non-HIV-exposed infants.^23^ Our data do not show more severe disease in children living with HIV or with TB disease but there was a relative high proportion of children who were identified with a new diagnosis of TB and two children with possible TB-IRIS. Both SARS-CoV-2 and *Mycobacterium tuberculosis* (*M. tb)* can elicit a hyperinflammatory state in the lung and the hyperinflammatory environment, induced by either *M. tb* or SARS-CoV-2 infection, could potentially accelerate TB disease progression or cause more severe COVID-19 disease.^24^ The exact role of viral co-infections in the inception, progression and severity of TB disease is unknown, and whether SARS-CoV-2 plays a role requires further investigations.

The COVID-19 mitigation strategies during the early phase of the pandemic initially led to temporary reduction in the occurrence of some other respiratory viruses.^25,26^ With the easing of non-pharmaceutical interventions such as lockdown measures and mask-wearing, respiratory virus infections increased.^27^ A relatively low proportion of children had viral co-infections compared to previous studies.^28^ This is partially because the Omicron wave occurred during summer in South Africa, with a general lower circulation of respiratory viruses. There was no evidence that viral co-infections were associated with more severe disease.

The interpretation of our data needs to be cognizant of some limitations. This study was conducted in a tertiary referral hospital and could therefore represent more severe disease. We did not include all cases of MIS-C seen during the Omicron wave; however, the role of Omicron in MIS-C cases in Cape Town have been previously described and clinical presentation and outcomes of MIS-C during the Omicron wave were similar to the previous three waves.^29^ Furthermore, we did not record COVID-19 vaccination coverage in the mothers of the children, which could potentially mitigate severe outcomes in infants. However, widespread SARS-CoV-2 seroprevalence was seen before the Omicron wave due to a combination of natural infection and vaccination immunity.^30^ Finally, due to changes in testing strategy, it is difficult to know how many people were infected during the Omicron wave and the estimated proportion of children and adults requiring hospitalisation.

In conclusion, the clinical presentation and outcomes of children during the Omicron wave in Cape Town were generally favourable, but severe respiratory disease was seen especially in infants and young children with underlying comorbidities. There was no evidence for more severe disease in children living with HIV or TB. Vaccination strategies for vulnerable young children need to be reviewed, especially in some African countries where there is not yet a policy for this age group. Children born premature might benefit from maternal vaccination during pregnancy.

## References

[1] Singhal T. The emergence of Omicron: challenging times are here again! Indian J Pediatr Published online 2022; doi:10.1007/s12098-022-04077-4. PMC8756165 35025038

[2] World Health Organization. Classification of Omicron (B.1.1.529): SARS-CoV-2 variant of concern. Geneva, Switzerland: WHO, 2021.

[3] Abdullah F, . Decreased severity of disease during the first global omicron variant covid-19 outbreak in a large hospital in tshwane, South Africa. Int J Infect Dis 2022;116:38–42. 34971823 10.1016/j.ijid.2021.12.357 PMC8713416

[4] Mohapatra RK, . Twin combination of Omicron and Delta variants triggering a tsunami wave of ever high surges in COVID-19 cases: A challenging global threat with a special focus on the Indian subcontinent. J Med Virol 2022;94(5):1761–1765. 35014038 10.1002/jmv.27585 PMC9015634

[5] Kitano T, . The differential impact of pediatric COVID-19 between high-income countries and low- And middle-income countries: A systematic review of fatality and ICU admission in children worldwide. PLoS One 2021;16(1 January):1–12. 10.1371/journal.pone.0246326 PMC7845974 33513204

[6] Nachega JB, . Clinical characteristics and outcomes of patients hospitalized for COVID-19 in Africa: Early insights from the Democratic Republic of the Congo. Am J Trop Med Hyg 2020;103(6):2419–2428. 33009770 10.4269/ajtmh.20-1240 PMC7695108

[7] Van Der Zalm MM, . Clinical experience with severe acute respiratory syndrome coronavirus 2-related illness in children: hospital experience in Cape Town, South Africa. Clin Infect Dis 2021;72(12):E938–E944. 33170927 10.1093/cid/ciaa1666 PMC7717210

[8] Hui KPY, . SARS-CoV-2 Omicron variant replication in human bronchus and lung ex vivo. Nature 2022;603(7902):715–720. 35104836 10.1038/s41586-022-04479-6

[9] Mahase E. Omicron sub-lineage BA.2 may have “substantial growth advantage,” UKHSA reports. BMJ 2022;376:o263. 35101887 10.1136/bmj.o263

[10] World Health Organization Pneumonia Vaccine Trial Investigators’ Group. Standardization of interpretation of chest radiographs for the diagnosis of pneumonia in children. Geneva, Switzerland: WHO, 2001.

[11] Harris PA, . Research electronic data capture (REDCap)―a metadata-driven methodology and workflow process for providing translational research informatics support. J Biomed Inform 2009;42(2):377–381. 18929686 10.1016/j.jbi.2008.08.010 PMC2700030

[12] Harris PA, . The REDCap consortium: Building an international community of software platform partners. J Biomed Inform 2019:95:103208. 31078660 10.1016/j.jbi.2019.103208 PMC7254481

[13] Goussard P, . Fatal SARS-CoV-2 Omicron variant in a young infant: Autopsy findings. Pediatr Pulmonol 2022;57(5):1363–1365. 35243813 10.1002/ppul.25881 PMC9088365

[14] Cloete J, Kruger A, Masha M, . Paediatric hospitalisations due to COVID-19 during the first SARS-CoV-2 omicron (B.1.1.529) variant wave in South Africa: a multicentre observational study. Lancet Child Adolesc Heal February 2022; doi:10.1016/S2352-4642(22)00027-X PMC8856663 35189083

[15] Murata Y, Tomari K, Matsuoka T. Children with croup and SARS-CoV-2 infection during the large outbreak of Omicron. Pediatr Infect Dis J 2022;41(5):E249. 10.1097/INF.0000000000003484 PMC8997017 35185142

[16] Martin B, . Acute upper airway disease in children with the Omicron (B.1.1.529) variant of SARS-CoV-2-A report from the US National COVID Cohort Collaborative. JAMA Pediatr 2022;176(8):819–821. 35426941 10.1001/jamapediatrics.2022.1110 PMC9012983

[17] Brewster RCL, Parsons C, Laird-Gion J, . COVID-19-associated croup in children. Pediatrics. Published online March 8, 2022; doi:10.1542/peds.2022-056492 35257175

[18] Tsoi K, . A child with SARS-CoV2-induced croup. Pediatr Pulmonol 2021;56(7):2377–2378. 33857338 10.1002/ppul.25408 PMC8251437

[19] Venn AMR, Schmidt JM, Mullan PC. Pediatric croup with COVID-19. Am J Emerg Med 2021;43:287.e1-287.e3. 10.1016/j.ajem.2020.09.034 PMC7490245 32980228

[20] Ludvigsson JF. Convulsions in children with COVID‐19 during the Omicron wave. Acta Paediatr. Published online February 10, 2022; doi:10.1111/apa.16276 PMC9303202 35098577

[21] Zhou Z, . Understanding the neurotropic characteristics of SARS-CoV-2: from neurological manifestations of COVID-19 to potential neurotropic mechanisms. J Neurol 2020;267(8):2179–2184. 32458193 10.1007/s00415-020-09929-7 PMC7249973

[22] Essajee F, . Child with tuberculous meningitis and COVID-19 coinfection complicated by extensive cerebral sinus venous thrombosis. BMJ Case Rep 2020;13(9):1–3. 10.1136/bcr-2020-238597 PMC7490923 32928816

[23] Le Roux DM, . Lower respiratory tract infections in children in a well-vaccinated South African birth cohort: spectrum of disease and risk factors. Clin Infect Dis 2019;69(9):1588–1596. 30925191 10.1093/cid/ciz017

[24] Visca D, . Tuberculosis and COVID-19 interaction: A review of biological, clinical and public health effects. Pulmonology 2021;27(2):151–165. 33547029 10.1016/j.pulmoe.2020.12.012 PMC7825946

[25] Sinha P, . Coronavirus disease 2019 mitigation strategies were associated with decreases in other respiratory virus infections. Open Forum Infect Dis 2021;8(6):2019–2021. 10.1093/ofid/ofab105 PMC8083776 34514014

[26] Marriott D, . Concomitant Marked Decline in Prevalence of Severe Acute Respiratory Syndrome Coronavirus 2 (SARS-CoV-2) and Other Respiratory Viruses among Symptomatic Patients Following Public Health Interventions in Australia: Data from St Vincent’s Hospital and Associated Screening Clinics, Sydney, NSW. Clin Infect Dis. 2021;72(10):E649-E651. doi:10.1093/cid/ciaa1256 32841316 PMC7499558

[27] Hodjat P, . The reemergence of seasonal respiratory viruses in Houston, Texas, after relaxing COVID-19 restrictions. Microbiol Spectr 2021;9(2):2020–2022. 10.1128/Spectrum.00430-21 PMC8557899 34494861

[28] O’Brien KL, . Causes of severe pneumonia requiring hospital admission in children without HIV infection from Africa and Asia: the PERCH multi-country case-control study. Lancet 2019;394(10200):757–779. 31257127 10.1016/S0140-6736(19)30721-4 PMC6727070

[29] Abraham DR, . The impact of SARS-CoV-2 variants on the clinical phenotype and severity of multisystem inflammatory syndrome in children in South Africa. Pediatr Infect Dis J 2022;41(12):e510–e512. 36102719 10.1097/INF.0000000000003691 PMC9645446

[30] Madhi SA, . Population immunity and Covid-19 severity with Omicron variant in South Africa. N Engl J Med 2022;386(14):1314–1326. 35196424 10.1056/NEJMoa2119658 PMC8908853

